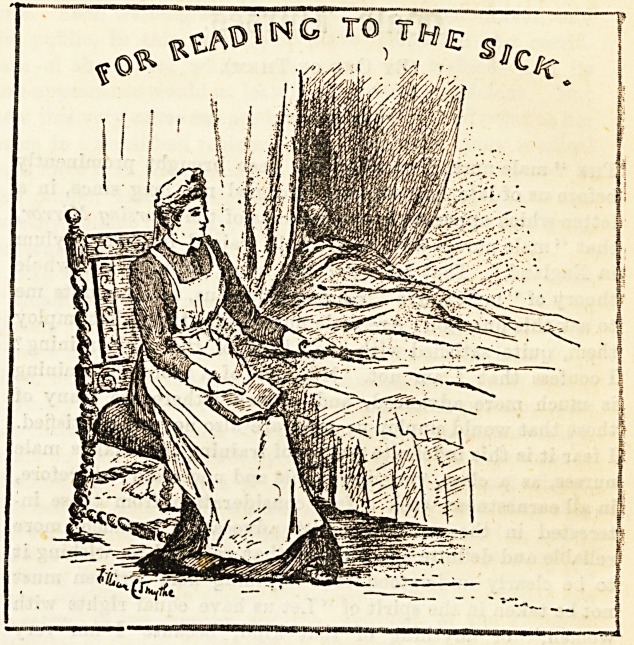# The Hospital Nursing Supplement

**Published:** 1891-04-11

**Authors:** 


### "The Hospital" Nursing Mirror.

Being the Extra Nursing Supplement of "The Hospital" Newspaper.

Contributions for this Supplement should be addressed to the Editor, The Hospital, 140, Strand, London, W.C., and should have the word "Nursing" plainly written in left-hand top corner of the envelope.

### En Passant.

St. Patrick's Home.—The annual report of this Irish institution for providing nurses for the sick poor is excellent. There is a staff of seven in the Home; a Superintendent, two nurses, two Queen's probationers, and two paying probationers. The Home has been affiliated to the Jubilee Institute, and to celebrate the occasion Mr. Rathbone, M.P., and Sir Edward Guinness each gave £100 to the funds of the Home.

Stewardesses. — Sometime ago a number of our correspondents were anxious to go as stewardesses, and common sense said that a trained nurse on a long voyage ought to make a very efficient stewardess provided she was a good sailor. Of the few who practically attempted the scheme we only heard from one who found it satisfactory. Quite lately one of our readers wrote: "I have got engaged on a Wilson liner, and I have been two trips to Hamburgh. I was very glad of the chance to go, but it is a dreadful life, and I shall be very glad to get back to nursing when my engagement is out."

For Prizes or Not?—The Lady Superintendent of an institution for nurses makes the proposal that we should offer a prize to the nurse who sells most copies of "Ministering Women." We are obliged to our correspondent for her very flattering letter, but we hardly approve her proposal. The book has been written to benefit the Royal National Pension Fund, and we are sure nurses interested in the Fund will do their best without the offer of a prize to spur them on. Besides, is not our feminine curiosity aroused to see the photographs in the book of those who have been instrumental in bringing the Fund to success? The prizes offered in this journal have never been of great intrinsic value—generally only some book as a mark of approval, a visible sign of appreciation for a piece of good work. We do not find that it is solely for the value of the prize so many nurses answer the Examination Questions, or make garments for us to distribute at Christmas.

Nuns versus Nurses.—A lecturer at Armagh lately gave some interesting facts concerning nuns and nurses during the Franco-German war. Herr Fischer, who was in the University at Berlin when the war broke out, and who left to join the Red Cross Society, mentioned that in one of the hospitals, during the progress of the war, there were a great many wounded. There was no cook and no female assistance. One day a knock was heard at the door, and presently half a dozen Protestant Sisters from Berlin entered. The Red Cross Men told them that they were in the greatest need of help from young ladies, and went on to explain to them that they would require them to sweep the rooms, cook, &c. The Sisters, however, said they had not been sent for that; that they only came to give spiritual consolation. At this the Geneva Cross Men got really angry, and said they could have plenty of spiritual consolation without them. The Protestant Sisters then went away, but shortly afterwards another knock came to the door, and some other ladies walked in. They said they were the Grey Sisters. The Geneva Cross Men told them what they wanted them to do, and the ladies replied, "Oh, that's the very thing we want to do. We have come to help in any way we can." They (the Geneva Cross Men) made them heartily welcome, and put them in the kitchen, and found them the best cooks, the best nurses, and the best ladies they had ever met.

Bravo Bristol!—The Duke of Edinburgh has agreed to play the violin and lead the orchestra at a concert to be given at Bristol on Wednesday, the 22nd inst., in aid of the District Nursing Society. The arrangements are in the hands of Mr. Riseley, and a most successful entertainment is expected.

A Pleasant Home.—To a nurse tired out with her hospital training we can imagine no more pleasant change than private nursing in connection with such a home as that at Hockerill, Bishop's Stortford. The situation is healthy, there is no over-work, and the nurses are paid from £25 a-year. The staff consists of a Lady Superintendent and four nurses, and the atmosphere of the whole place is cheerful yet restful. We were not surprised to learn that nurses who once join this home work for it for years.

A Blind Masseuse. — At the instigation of the Charity Organization Society, Miss Annie Chamberlain, a blind girl, has been trained by a medical man as a masseuse. Everyone knows how sensitive is the touch of a blind person, and it is found that certain nervous patients prefer the services of a rubber who cannot see. If this step widens the field of employment for those deprived of sight, it will be an excellent thing. Some time since we heard that Mrs. Creighton Hale had offered to teach massage free of expense to two blind girls.

The Nurses' Co-operation.—The first quarterly meeting of the Nurses' Co-operation was held, on Tuesday, at 8, New Cavendish Street, Mr. Charles Cheston in the chair. It was announced that over 300 applications had been received during the three months, and that 91 nurses had been enrolled. It is not proposed to enroll any more nurses until next year. The report of the Finance Committee was presented by the hon. Treasurer, Dr. James A. Goodhart, and unanimously adopted. It was agreed that the support of the medical profession should be widely asked for this movement, since its object is to secure to fully-trained nurses the full remuneration for their work.

Short Items.—The Mercers' Company have given £21 to the Workhouse Infirmary Nursing Association.—Last Saturday's *Truth* contained a long article headed, "St. Bartholomew's Must be Closed," whereas the *Queen* contained a notice from Miss Isla Steward that more nurses were wanted to enter at once.—Sheffield Guardians have repented, and appointed a Lady Superintendent to the Infirmary.—Stirling is to have a Queen's nurse.—Miss Thompson, Lady Superintendent, sends us a summary of the cases nursed at the Barnstaple Nursing Home during the first year of its existence. The list is long, and the Home must have supplied a great want.

Paddington Green.—We have received a long letter from Mrs. Phillips, of Norwood Cottage Hospital, in which she declares that she finds the answers of the committee of Paddington Green Children's Hospital to her complaints printed in The Hospital of January 24th, to be unsatisfactory. We have again applied to the Secretary of the Hospital, who has courteously given us further information. Mrs. Phillips is entitled to all sympathy for the sad loss of her niece, but her grievances have been met, and we cannot see that any good can ensue from continuing the correspondence. It is not as though the Committee of the Children's Hospital refused to hearken to Mrs. Phillips, or to make such alterations as circumstances proved necessary, but now Mrs. Phillips is bent on widening the issue and including points on which her information is not correct. We cannot, therefore, admit the controversy to our pages.

### Lectures on Surgical Ward Work and Nursing.

By Alexander Miles, M.B. (Edin.), C.M., F.R.C.S.E.

### LECTURE XX.—SPECIAL CASES (*continued*).

Tracheotomy.—This operation of opening the trachea or wind-pipe, and inserting into it a tube through which the patient breathes, is usually performed for croup or diphtheria, although it is sometimes required for other conditions. The success of the proceeding depends as much on the after treatment as on the performance of the operation itself. After the operation the patient is put into a bed surrounded by a tent, readily extemporised by screens and blankets, and the air in this is kept moist by setting one or two bronchitis kettles by it. The temperature inside the tent should be about 70 deg. Fahr., and draughts are to be studiously avoided. The child must be fed frequently and regularly with stimulant food, such as brandy, Valentine's meat juice, etc. ; and should the heart show signs of failing, strophanthus or digitalis may be added. The cleaning of the tracheotomy tube is the most important part of the nurse's duty. The tube, which is usually made of silver, is double, the outer one being tied into the trachea, while the inner one is loose, so that it may be removed and cleaned, and also that, should it get blocked, the patient can cough it out, and so breathe through the outer one alone. At first the inner tube should be removed very frequently, say every twenty minutes, and washed in carbolic lotion, the lumen being conveniently cleared with a feather. The excess of carbolic lotion should be dried off and the tube lubricated with glycerine before being reintroduced. One or two layers of gauze spread over the mouth of the tube filters the air and prevents the inhalation of dust, etc. The tube is left in "as long as it is required," a point which will be settled by the surgeon.

Caution.—The nurse must take every precaution against herself being infected with the disease from which the patient is suffering. To this end she must avoid unnecessary handling of the child, especially kissing it, and, above all, should never get into direct line with its breath when coughing. She should frequently gargle her throat with Condy's fluid, or some such antiseptic, and, of course, should never take food in the patient's room. There is one great danger peculiar to cases of diphtheria of which I must warn you, and that is the sucking of the tracheotomy tube. When this gets blocked, and all your attempts with the feather fail to clear it, the temptation to relieve the great distress of your patient by sucking the tube is very great. Such a procedure, however, is quite irrational. Consider for a moment the condition of matters before you. The false membrane is blocking the air passages and diminishing the amount of oxygen getting into the lungs to aerate the blood—in other words, the patient's distress is due to the small amount of air in his lungs. What is the effect of sucking the tube? First you withdraw the little remaining air from the patient, and if your sucking power is sufficiently strong, you may succeed in getting out some of the obstructing matter. As a rule, however, you do not, and you leave the patient worse than you found him, and, not only so, but you have run an enormous risk, a risk almost amounting to a certainty, of being yourself infected. Not a few valuable lives, both of nurses and surgeons, have been lost in this way, and although the devotion to the interest of the patient, which prompted the action, is much to be commended, the foolhardiness of it is certainly not to be emulated.

Operations on the Mouth and Jaws.—The principal duty of the nurse in cases of this nature is—(1) *The feeding of the patient*, as much of the success of the operation depends on his being able to take a sufficient amount of nourishment to carry him over the early days. As his power of masticating is gone, and of swallowing much impaired, it is usually necessary to administer his food partially digested through a stomach tube, passed either through the mouth or nose. In *passing the stomach tube* the patient is directed to hold his head well back, and to open his mouth wide. The forefinger of the left hand is passed far back into the pharynx to guide the tube, which has previously been lubricated with glycerine, past the epiglottis. When the end of the tube has reached the back of the mouth the patient is asked to swallow it, and as he does so it is gently pushed on, the head at the same time being brought into its natural position again. Care must be taken before introducing any food that the tube is not in the larynx or trachea. When it is so, in addition to the coughing of the patient, the sound of the air passing through the apparatus is sufficient to warn you. The patient is fed with peptonised milk, Valentine's meat juice, switched eggs, brandy, and so on; if necessary, supplemented with nutrient enemata. (2) *The mouth must be kept as clean and aseptic* as possible by frequently washing it out with Condy's fluid, or boracic lotion. This may be done by a small sponge *securely* fixed to a wooden handle, or held in long forceps ; or, better still, by a syphon arrangement fixed over the patient's head, so that a stream of lotion may be allowed to run in at one angle of the mouth, and out at the other. Keeping the mouth sweet is of great importance for several reasons, (*a*) it is very comforting to the patient, (*b*) it diminishes the risk of septic absorption and consequent septicæmia, and (*c*) it diminishes the risk of septic pneumonia, a very frequent cause of death in these cases. (3) When the lower jaw has been excised, *the tongue* looses its anterior attachments, and *is liable to fall into the back of the mouth* and block the air passages. Should this occur the patient's head must be turned on one side, or even face downwards to permit of the tongue falling forward again. If this be not sufficient, it must be seized with catch forceps and forcibly pulled forward. (4) Reactionary or secondary *hæmorrhage* is a source of danger in tongue cases. The nurse must be prepared to pass her finger well back into the mouth, and to press on the bleeding point, while *some one else* goes for the doctor.

Abdominal Operations.—These usually come under the care of special surgeons, and of nurses trained for the work. One or two points, however, may be mentioned. The main indications in the after treatment are to give the bowel absolute rest, to avoid sickness and vomiting which may cause bursting open of the wound in the abdominal wall ; and above all to ensure the most perfect asepsis. To ensure the first two objects the patient must be kept on very low diet for the first few days after operation. After being put back to bed she should have nothing for the rest of that, and all the following day, save a few sips of tepid water, simply to allay her thirst. Should there be sickness the water may be given cold. At the end of forty-eight hours of this regimen the diet may be gradually increased, milk and potass, Valentine's beef-juice, chicken tea, light puddings, etc., being in turn given. At the end of a week, should the bowels remain closed, a gentle purge aided by an enema may be given.

### Presentation.

Staff Nurse Chappel, of the Royal Infirmary, Edinburgh, has been appointed Matron of the Montrose Asylum. Before leaving Edinburgh, Nurse Chappel was presented by the clerks of Professor Greenfield's wards with a handsome carriage clock, a book on mental diseases, and a beautifully illuminated address.

### Death in Our Ranks.

We regret to announce that Sister Roberts, of the typhoid wards at the Bradford Fever Hospital, has passed away after a long and painful illness. She served 18 years of faithful service, and was greatly respected and is deeply regretted.

### The Eastern Hospital Scandal.

The inquiry into the alleged maladministration of the Eastern Fever Hospital was resumed on April 2nd, at Norfolk House, Strand. Matilda Basham said that from 1877 to 1888 she was a nurse at the Eastern Hospital, and remembered the incident of a child that was supposed to have died from drinking carbolic hair-wash, in June or July, 1888. When she heard of it she sent to Dr. Bence, who came and gave the child an emetic. There was no foundation for the suggestion that it had taken carbolic acid. While she was at the hospital the food, generally speaking, was good. The milk sometimes turned sour, but not often. In cross-examination the witness stated that Dr. Collie was very angry about the incident of the alleged carbolic poisoning, because he was always very particular to have all poisons locked up. Occasionally patients complained about the diet, and the doctors altered it. Lydia Weston said that when the nurses saw a letter about the hospital in a local paper they were generally indignant. Cross-examined: Miss Dowsett told her to draw up a letter, which was signed by the nurses. In re-examination witness stated that Nurse Halkin's letter to the local paper was untrue. Miss Emily Aston said she was now Matron at the Colonial Hospital at Gibraltar, and had come from Gibraltar to give evidence. She entered the Eastern Hospital as Matron on the 14th of June, 1887, and left on the 15th of February, 1890. Speaking generally, the quality of the food was very indifferent; the milk was sometimes sour; and she had been spoken to by the nurses about it. There was a dietary table hanging up in most of the wards. Upon the table the fish was described as "cod, sole, or brill," but she never saw any of these supplied to the patients. The fish was mainly haddock and other coarse kinds. What do you say as to the discipline at Homerton?—I think it left much to be desired. Did Dr. Collie's conduct assist in the maintenance of discipline among the patients?—No. Taking all things into consideration, I don't think it did. In what way?—Well, I refer more especially to the dance in Faith Ward. I don't think his conduct then——Mr. Gye objected to this evidence. Mr. Hedley said that as Nurse Sara had referred to the dance the evidence was admissible. The witness, in answer to further questions, said that Dr. Collie's remarks in the presence of the nurses were not conducive to discipline. Once she heard him make reference to changing the names of the wards. Dr. Collie then referred to the committee as "swine." There were two nurses present. It was suggested that the wards should be called after saints—(laughter)—but Dr. Collie said they should be called after some of the committee—John, Isabel, and Maud. On one occasion we were discussing the steward, when Dr. Collie said the steward was a fool, and that anyone could see that directly he raised his hat. (Laughter.) There was no room for brains, and he was all lower jaw and voice. (Laughter.) I remember that in 1889 two of the assistant nurses resigned in consequence of the bad quality of the food. I remember also that some of the bedding in the diphtheria ward had a very bad smell, and I advised the assistant nurses to call the attention of Dr. Collie to it; but I do not remember that anything was done about the matter. It was a rule of the hospital that nurses should change their clothing before going out, but I ascertained that many of them did not, and I represented the matter to Dr. Collie. I had no opportunity of seeing how things were disinfected at the hospital. I have known of "raffles" being arranged in the hospital by the night superintendent. I don't think a young woman ought to be appointed assistant nurse under the age of twenty-two. I resigned my position as matron in consequence of Dr. Collie ignoring my authority in connection with the dance among the nurses on Twelfth Night in 1889, in the Scarlet Fever (Faith) Ward. I discovered after the dance that the nurses went out of the fever ward into the enteric and diphtheria wards without changing their garments. At this stage the inquiry was again adjourned.

WARNING.

Health is among the greatest of God's gifts to man. What pleasure it is to be able to walk and run and buffet with the strong breeze, or to do our daily work with ease and comfort, feeling only such an amount of fatigue when we leave off, as will make the rest we have earned by our exertions the more enjoyable? It is, indeed, an inestimable blessing, but how seldom we value it, till we are nearly losing it, or it has slipped from us past recall. We look on health as our birthright, and so small ailments are sent, to teach us not to abuse our strength but to take care of it and use it with grateful hearts. A sprained ankle, the flesh sore with bruises, or the joints and muscles nipped with rheumatic pains, startle our security and remind us that sooner or later we "must shuffle off this mortal coil" and return to the dust from which we sprang.

You who are sick and suffering have hope while there is life? God is very good and seldom takes away our powers with a stroke unless we have used them recklessly, but chastens us to show we are His beloved children and gives us time and opportunity for self recollection, lest we meet death unprepared.

The old fable of "The three warnings," has an excellent lesson and a true moral. Death says Æsop claimed a young man in the height of health and prosperity. "I am not ready to go," said the poor fellow, "it is very hard to tear me from my bride, my riches, my pleasures just now, you might at least have given me fair warning of your approach." "If I had done so you would have taken no notice of it," replied the other. "O! spare me this once," cried the sick youth. "I will be quite ready the next time you come if I have due notice."

So the King of Terrors left him for the time and more than three score years and ten had passed ere he again made his appearance. "You here so soon again!" exclaimed the man. "I should have been quite prepared had you kept your promise of warning me of your coming, but I have received none." "Ah!" rejoined Death, "it is as I foretold, you would not take the hints I sent. Ten years since your sight failed you, that was the first sign I made; next you became deaf, and you did not recognise my footsteps drawing near; now you cannot walk without help, why then reproach me for my neglect?"

"Spare me yet a few years—a few months—a few days even" he moaned, but the grim messenger was inexorable and laid his cold hand on the aged man.

Your present sufferings are a reminder that you have a soul as well as a body to save; number then, your days and apply your heart unto wisdom so that when your appointed time is come you will find death but an angel of light who will guide you to a blessed eternity.

### Male Nurses.

(By One of Them).

The "male nurse" question has been brought prominently before us of late, and we were informed not long since, in a letter which appeared in the columns of the *Nursing Mirror*, that "male nurses were thoroughly trained in every asylum in England." That letter, to my mind, brought the whole theory of "male nurse" training before us, and prompts me to ask this question: Are male nurses, and those that employ them, quite satisfied with that kind of thorough training? I confess that I am not. My idea of a thorough training is much more advanced, and probably there are many of those that would employ us that are also not quite satisfied. I fear it is this indefinite system of training that makes male nurses, as a class, very unreliable and uncertain; therefore, in all earnestness, seek a fair consideration from those interested in the matter, whilst advocating a much more reliable and definite system than that referred to, wishing it to be clearly understood that anything here written must not be taken in the spirit of "Let us have equal rights with women," or anything of that kind, because I am very decided and strongly of opinion that actual nursing is a woman's vocation, and that they alone are fitted for and equal to general nursing, but the spirit in which I advocate is rather—men are absolutely necessary as nurses in special cases of male nursing, whether they be trained or not, therefore for these admitted necessary cases let them be as perfect as possible, and let us consider whether necessary and reasonable facilities cannot be granted that they may train in the wards of our hospitals, and obtain certificates to show that they are so trained and perfected. It is a well-known fact at the present time that the good and the bad, the trained and the untrained, are all so thoroughly intermixed that male nursing, as a whole, cannot fail to be unreliable and unsatisfactory to all concerned. It is the chief cause of the many scandals that are brought before us from time to time, we may be assured, as it also is of the slurs that male nurses meet with and are liable to during the course of their employment. Of course, we must all gladly acknowledge that we have many good and valuable male nurses, good in every sense of the word—well trained, conscientious, reliable, honest, and gentle—and it would be hard indeed to better them. They receive very good training, and experiences from various sources, especially so from our naval and military hospitals, and more or less so from our public hospitals and public and private asylums. Nevertheless, the flaw exists in not having a recognised and definite system of training, in my opinion, and also in the want of a certificate when trained. These wants leave it open for unsuitable men being employed as "male nurses," who are in no way fitted to be so employed, and who are sadly too often bringing discredit upon those that are so fitted. Perhaps, by way of illustration, I may be allowed to show from experience how this want of a recognised system of training affects male nurses and their employment in some of the provincial towns. Amongst ten or twelve men employed from a "private nursing institution," kept entirely for the profits it makes for the proprietor, sometimes not more than one or two men, by any stretching of the words, can be called male nurses. I have known a man entered for employment with nothing further to recommend him than that he was of fairly good character, and had had six weeks' satisfactory employment as an attendant on an invalid. These men receive a yearly pittance and board when not on a case. The majority of them are not worth anything as male nurses, for they are utterly unfit for the work, and never ought to be employed as such. The supply is unlimited. Advertisements often appear from such institutions offering every inducement to female hospital-trained nurses to join their staff. I have even seen them go so far as to offer them all their own earnings, but I should think there was some trickery when they did that, otherwise it seems strange that they would board and lodge them when not out at a case. But what a difference if a "male nurse" is wanted. Commonly enough, then, this expression may be heard: "Oh, we have only to advertise in the local newspaper, and they will come. They are flocking here from all directions, and our only difficulty is to select who may be most useful." Yes, most useful, not "who may be best trained," or "most reliable," for that is not the point considered. May we not fairly ask, Why this difference? They are all wanted for nursing the afflicted. It is simply because of the females being trained, and possessing certificates in proof of being so, for, be it remembered, in these days it is hardly thought safe to send out females as nurses that are not properly hospital trained. Most of the men are not trained, properly or improperly, and it must too often be thought that it is not necessary that they should be. The Hamilton Association of London are pioneers, advancing slowly but surely. The careful method they adopt in selecting candidates for admission, and better still, the endeavour they make to train their own nurses, are bound in the end to tell for good. Whilst proudly looking forward to a well trained, competent body of male nurses, and proper and general facilities for training such, perhaps we may prudently discuss the question, Should male nurses wear a uniform? It is a very open question whether they should or not, and much could be brought forward on either side. I find that I rather incline toward "no uniform," yet I cannot see any very great objection to their wearing a uniform, provided it is made as neat, simple, and plain as possible. The strongest objections may be offered against anything either showy or conspicuous, and if even male nurses do have a uniform, it will be nearly impossible to lay down any hard and fast rule about their wearing it. Probably male nursing cannot well be separated from a great deal of attendant work, being part of their duties. Some better authority will perhaps enlighten us on this point. In private work it cannot well be done, and supposing that it cannot generally, it occurs to me that it will always be a question for our employers to decide whether they would care for those they employ being in uniform or not. Again, friends of slight mental and epileptic patients, and others requiring out-door exercise, would have to be considered, and objections of that kind present themselves and have to be met. Then, again, an extra stock of clothing would have to be kept ready for cases of emergency, or otherwise the nurse must be sent out in uniform, whether those employing him wished him so or not. There cannot be much objection to a quiet, plain uniform, indoor and out, whilst training or whilst at actual nursing, and perhaps there is much to commend a recognised indoor uniform, especially in nursing infectious cases, but on the whole—and being of opinion that it would be much better to train men that had been employed as attendants only into being male nurses, and thus qualify them for all cases, rather than have separate classes, one for nursing and the other for attendant duties—I incline rather toward no uniform, with this qualification, that I would not care to give a decided opinion until I had heard the views of those who superintend a body of male nurses, as they would be likely to judge the advantages both for and against, rather than one at individual work. A uniform would be a badge of one's training, and I very readily admit that would be an enormous advantage; but evidently the question is an open one, and I should like to get some expression of opinion from my fellow nurses.

(*To be continued.*)

### The Register of Nurses.*

It will be within the memory of most of our readers that we have persistently declared that the time for the registration of nurses is not yet come; and that the attempt made by a few to force this movement forward was bound to be a disastrous failure.

Our reasons for this conclusion were obvious; "training" in nursing has only existed for 18 years, and has only been common for the last ten; so that the number of properly trained nurses only numbers about 5,000 in the United Kingdom, while the number of those employed as nurses is probably from 15,000 to 20,000. Evidently then nursing is still in a transition state, and any attempt to legalise it as a profession had better be delayed till the trained nurses, out-numbering their untrained sisters, shall have made the standard of nursing worthy of general recognition.

But in spite of the fact that these opinions were expressed by the leaders of the chief training schools, the movement was hurried precipitately forward. The notion of a Royal Charter was indeed abandoned, and the present attempt to secure incorporation under the Board of Trade seems likely to fail. Still, there has been published what is called "The Register of Trained Nurses," and it is of such a character that we feel bound in the interests of all good nurses to subject it to criticism.

First, we note with surprise the names of the Registration Board, and we deeply regret that men of such undoubted honesty and with such a high sense of honour as Mr. Brudenell Carter, Dr. Sainsbury, and others, should permit their names to stand on the opening leaf of this volume.

Next we note the Preface; and we would ask our readers to remember that it states that the object of the Register is to prevent a nurse guilty of "drunkenness, theft, or graver offences," from continuing her professional duties under cover of a certificate. It is important that this phrase should be remembered:—"*Drunkenness, theft, or graver offences.*"

On the very first page of the Register we find the name of a nurse who had to leave her hospital for "having in her possession an article belonging to another probationer."

This nurse holds no certificate from her training school, and had sufficient inquiries been made, or proper references demanded, her character would have been discovered. We are obliged to conclude that no adequate inquiry into the characters of the nurses on the register has been made, and we would demand of the registration board whether they consider they had no duties in this matter? Doubtless not; they gave their names out of sheer good nature and sympathy with nurses; but surely never were good nature and sympathy so out of place! Some one has got to be responsible for this Register. It is no pleasure to us to rake up old nursing scandals and expose our sores, as it were, to the eyes of the public; but such a volume as this it is impossible to pass over in silence, for it is sure to mislead. Note this very first example: a nurse, leaving her hospital for a most serious fault, is put on this Register, and under the regulations we read "6.—Every nurse who is registered will receive a certificate under the seal of the association." And yet this is the register which was to remedy the abuse of a nurse guilty of "*drunkenness, theft, or graver offences,*" continuing her professional duties under cover of a certificate!

The training schools, by this one example alone, can prove how right was their contention that the register at the present time would simply increase the evils it pretended to cure. Each training school registers its trained nurses, and the public, in this case, could have demanded the certificate of the school where this nurse was trained—and its non-appearance would at least have aroused suspicions. But now this very nurse can produce a certificate, and point to her name in a published register; and seeing how many medical men have lent their names to the register, can we wonder if the public, unless enlightened, should consider it a trustworthy volume? Truly, this is a peculiar way of remedying present abuses!

Still keeping to the first page, we find under the head "Qualifications, Training, &c.," the following: "Cert., Queen Charlotte's Hosp. in 1880; London Association of Nurses to date." Here we must turn to a side issue for a time, but a very important one. Amongst the many extraordinary things told to the Lords' Committee perhaps none astonished the audience of nurses more than statement 9,434, made on July 28th, 1890, by Miss Sprigg, with reference to the London Association of Nurses, 123, New Bond Street. She said: "They (the nurses) are not accepted unless they have had three years' hospital training to begin with, and a careful study of the register shows that the average length of training is nearly four years." Now, take the case of the nurse certificated at Queen Charlotte's: that certificate can be earned in two months, and the certificate of the Lying-in Hospital in Endell Street can be obtained in one month, and that of the City of London Hospital in three weeks. Now, according to "The Register of Trained Nurses" there are of those members of the London Association who have gone on the register, as set forth therein, 40 who received less than the three years' training, Miss Sprigg's assertion notwithstanding; of these 40, as stated in the Register, 12 have had only one year's training; 10 have had only lying-in training; and 5 have had only asylum "training"—if such a thing exists. On another occasion we may continue our examination of "The Register of Trained Nurses;" meanwhile, we would ask all nurse training schools to compare their register with this published register, and to let us know (1) what percentage of their good nurses are on the published register; (2) the names of unsatisfactory nurses who are on the published register.

* The Register of Trained Nurses for 1891: 8, Oxford Circus Avenue, W. 2s. 6d.

### Notes and Queries.

### Queries.

(1) *Publisher Wanted.*—The name and address wanted of the publisher of "Our Daughters," by Dr. Oapp.

(2) *District Nursing.*—Wanted the addresses of district nursing societies not entirely free—the patients making some small payment.—*D. N.*

### Answers.

(43).—Raw clean potato is a most excellent dressing for a burn. I have used it with great success, though I am a nurse. Scrape a raw potato to a pulp, mix with lucca oil, and apply.—*A. G. F.*

(45).—Can air and water beds be mended? Certainly. Those belonging to the North London Nursing Association are mended by Benson, Upper Street, N., with india-rubber solution. A bed beginning to wear will stand several mendings.—*K. S. M.*

(46)—Nurse De Levante recommends Miss H. two unfurnished room in the house she is living in. Please address Mrs. H. Phillips, 37 Herbert Road, Stockwell, S.W.

*E. B.*—We have no room to print your lines.

*C. M.*—No matter what your age you are eligible for your share Bonus Donation Fund.

*Nurse Miriam.*—Get "Lectures on Massage and Electricity," by Stretch Dowse. Published by Wright, of Bristol, price 7s. 6d., post free.

*District Nurse.*—At Guernsey, Channel Isles, Richmond, Surrey, and elsewhere, there are corps of ambulance pupils who help in district nursing. The Secretary of the St. John's Ambulance Association could give you all particulars.

*Nurse Murray* begs to thank whoever is sending her THE HOSPITAL, Postmark, Lancaster.

*M. G.*—We shall be glad to use the lines "Toil On" when we have room.

*Mars.*—Write to or call on the Matron of the nearest hospital; or get a copy of the "Hospital Annual," where you will find all about how to become a nurse.

*Nurse.*—Get Barnes' "Manual of Midwifery," or if you want something cheaper and do not mean to take up monthly nursing, get "New Life," published by Swan Sonnenschein.

*A. C. and Others.*—Next week; our columns are so full.

*C. S. K.*—We will admit your paper for competition. Thanks for your kind letter.

*Miss S.*—Nurses' cloaks can be had ready-made from Messrs. Shoolbred, Tottenham Court Road, W.C. They supply the pretty cloaks of the Charing Cross nurses. Price about 18s.

### Buzzing of a "City Bee."

As I wing my way to and from the great City hive of busy workers, I have to cross and re-cross with the surging stream on one of our City bridges, and am invariably met by some of those plain, but becomingly dressed women that denote the wearers to be nurses in one of our hospitals. Thus am I reminded that amid the striving and struggling of our City life there is silently going on in our midst the almost unremarked but great and good work that is fighting death in its manifold form of approach, and mitigating suffering, or restoring newness of life to the bruised and maimed in the ever waging conflict.

As they pass, there often seems a gleam of brightness illumining the face, as though a temporary release from those wards where pain and sickness are ever present and contact for a while with the outer and more vigorous activities of life, was to them the advent of a new strength and refreshment for the work to which they have given themselves, and one may be justified in feeling something of admiration, something of more than ordinary interest in women who engage with such devotion and self-sacrifice in the work to which they have given themselves. Surely they are most deserving of our sympathy and support. I can imagine such an one leaving for a while the couch of some poor sufferer, the glance of whose eye, the touch of whose hand is sadly missed, and who is rewarded with no little impatience, as the ministering angel to that patient, whose place can hardly be supplied by another, although equally gentle and kind.

And yet from the very frequency with which we meet these forms that every now and then come across our path, how few of us pause to realise some of those great facts that are suggested by that neat attire? Shall we think of the vast number of them in all our nursing and benevolent institutions, each differentiated perhaps by some slight variation of dress? Shall we think of the patience, the self-denial, the training that precedes the activities of the work? That not a few of them are from the ranks of those who could have lived in ease and comfort, but who, with a noble self-surrender, have preferred to minister help and comfort to those in need of it, while others not so favoured have chosen it as a profession honourable to themselves and useful to others?

But, of course, if the underlying motive of self-sacrifice has prompted and also animates the worker, if right and holy principles are the groundwork of the whole, there can hardly be found a field for philanthropy so wide as that upon which such women enter; and if they themselves are of the true stamp (such as we are disposed to think them), then we must render a just tribute to such women and long to know more of their great unobtrusive work.

Ere the Busy Bee has time to gather up such thoughts he is involved in the great hum and bustle of the City hives, and perhaps for a while the stream of daily cares and duties shut out reflections like these, and he has to "buz" to a very different tune; but the morrow comes, and he may be brought back again to think not only of the workers, but the spirit of their work.

It seems to him that there is in the character and spirit of our various institutions, and especially our hospitals, a grand testimony to the great superiority of the religion of Jesus Christ to all others. Why this very humane development of it is really divine. What in all the other schemes of men can be shown to compare with it? Socialism talks, but Christianity acts. Till He came, who went about doing good, who ever thought of the wants of suffering humanity? The very lepers, driven to dwell among the tombs, were instances of the exclusiveness of the charity of those times. But when He came even these were healed by His touch. No careworn pain-stricken face was ever upturned to Him without exciting His tenderest pity and securing His gracious aid. Promptly came the healing word, or the "virtue went out of Him" to meet the suppliant ere the prayer had reached His ear. If I wanted to gather the "evidences for Christianity" I should certainly class among them our hospitals and other institutions with their voluminous facts that tell with irresistible force of the mind that was in Christ Jesus as the Great Physician of a world's woe and sin. It therefore seems to the "Busy Bee" that an irreligious worker is an anomaly here, and he greatly prefers to think of them all as co-workers with God, as all good and true, and so he would hum out some cheerful notes to such that they be not weary in well-doing, bidding them remember that only in ceasing to live to ourselves do we become personally enriched and blessed.

### Appointments.

Bristol General Hospital.—Miss Caroline Fishwick has been appointed Night Superintendent at this hospital. Miss Fishwick trained at the London, and was also Staff Nurse there for a few months, after which she was eleven months at King's Lynn.

Govan Parochial Hospital.—Miss Flora Macdonald has been appointed Matron of this hospital; she was four years at the Glasgow Western Infirmary, and has since worked on the staff of the Hillhead Nurses' Home. Miss Macdonald holds good certificates and testimonials.

Maryland University Hospital.—Miss Louisa Parsons has been appointed Superintendent of the Training School for Nurses connected with this hospital at Baltimore. Miss Parsons trained at St. Thomas's, worked through the Egyptian campaign, and then went out to America to join the nurses of the John Hopkins Hospital.

Newcastle Royal Infirmary.—Miss Agnes Ross has been appointed Lady Superintendent of Nurses to the Newcastle Royal Infirmary.

Royal Bath Hospital, Harrogate.—Miss A. A. Gwyn (who trained at the Hants County Hospital, Winchester, and subsequently worked at Victoria Hospital, Netley, and St. Bartholomew's Hospital), was appointed Matron of this institution (125 beds) on the 9th ultimo. She was formerly Accident Sister at the Hants County Hospital, holding successively the office of Matron (*locum tenens*) to the Radcliffe Infirmary, Oxford, and Matron of the General Hospital, Birmingham, and General Infirmary, Worcester, respectively. Miss Gwyn has excellent testimonials, and we wish her every success in her new position.

### Amusements and Relaxation.

### SPECIAL NOTICE TO CORRESPONDENTS.

**Second Quarterly Word Competition commenced April 4th, ends June 27th, 1891.**

Competitors can enter for *all* quarterly competitions, but no competitor can take more than *one first prize* or *two* prizes of any kind during the year.

The words for dissection for this, the SECOND week of the quarter, being "SAN REMO."

| Names. | April 2nd. | Totals. | Names. | April 2nd. | Totals. |
|---|---|---|---|---|---|
| Reynard | — | 77 | Madame B. | — | 25 |
| Reldas | 76 | 682 | Shakespeare | — | 59 |
| Tinie | — | 30 | Smyrna | — | 259 |
| Patience | — | 76 | Southwood | — | 102 |
| Jenny Wren | 63 | 594 | Gipsy Queen | — | 21 |
| Agamemnon | — | 596 | Snowball | — | 19 |
| Wyameris | — | 391 | Rita | — | 88 |
| E. C. | 67 | 654 | Mortal | — | 16 |
| Ecila | — | 283 | Nurse Annie | — | 15 |
| Hope | 79 | 680 | Carmen | — | 11 |
| M. W. | — | 583 | Grannie | — | 45 |
| Qu'appelle | 61 | 644 | Amie | — | 30 |
| Nil Desperandum | 72 | 667 | M. R. | — | 25 |
| Lady Betty | 69 | 621 | Primrose | — | 24 |
| H. A. S. | — | — | Nurse J. S. | 60 | 409 |
| Sister Jack | — | 62 | B. A. C. | — | 223 |
| Crystal | — | 203 | Theta | — | 148 |
| Woodbine | — | 25 | Ugug | — | 51 |

**Results of First Quarterly Word Competition.**

*First Prize*, 15s., is awarded to Reldas (Miss Alice Sadler), 32, Norland Square.

*Second Prize*, 10s. is awarded to Hope (Miss Mason), 112, Mildmay Road, N.

*Third Prize*, 5s., is awarded to Nil Desperandum (Miss Alice Meadows), Royal Hospital, Putney.

## Figures and Tables

**Figure f1:**